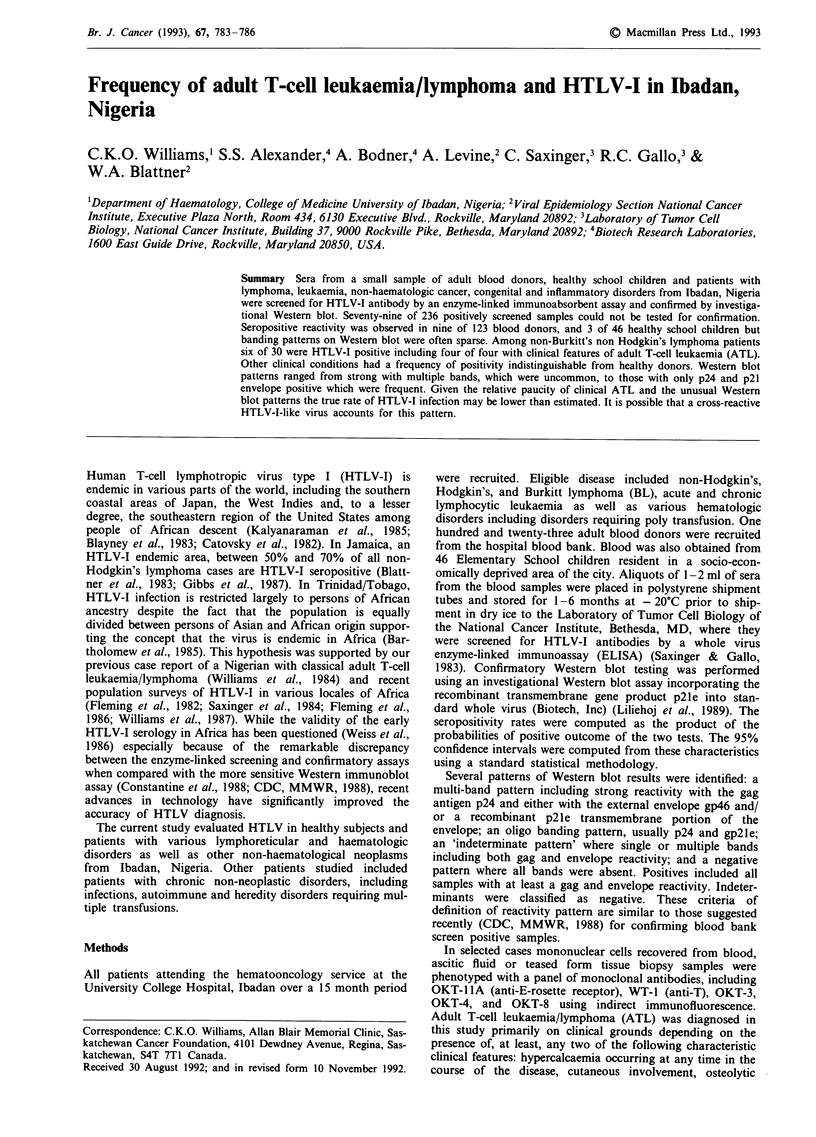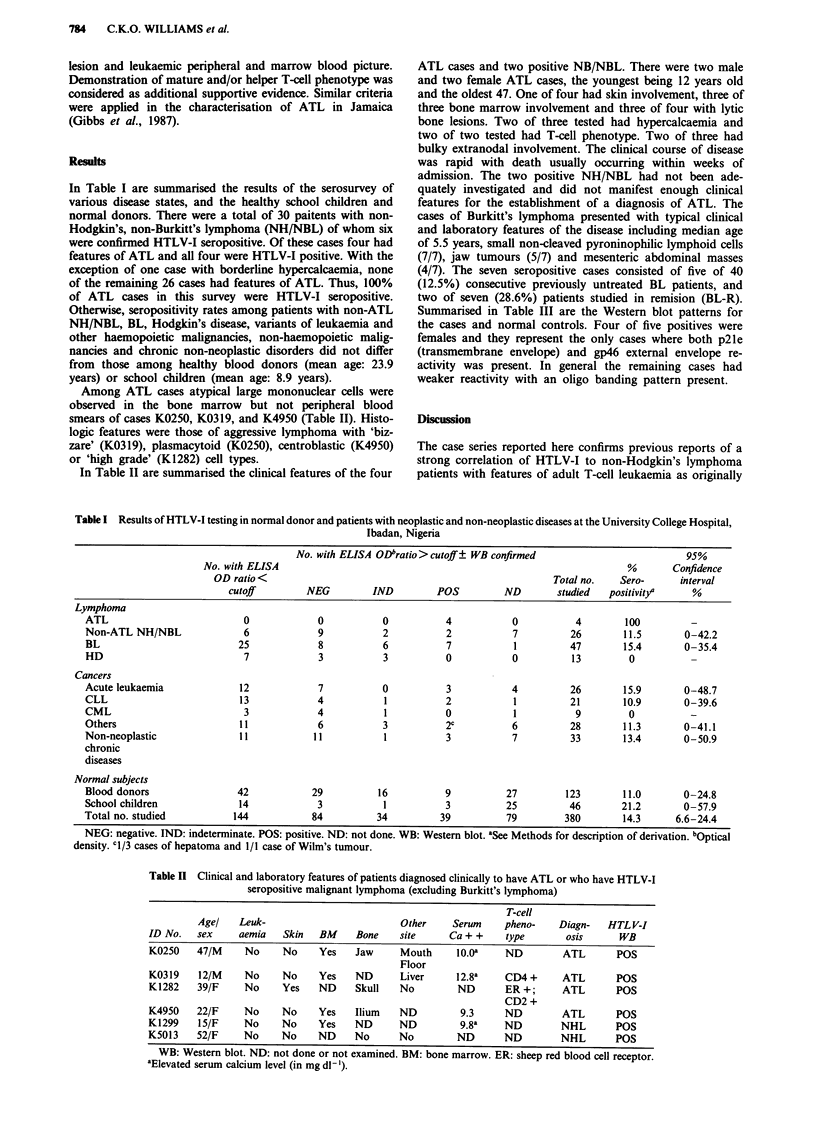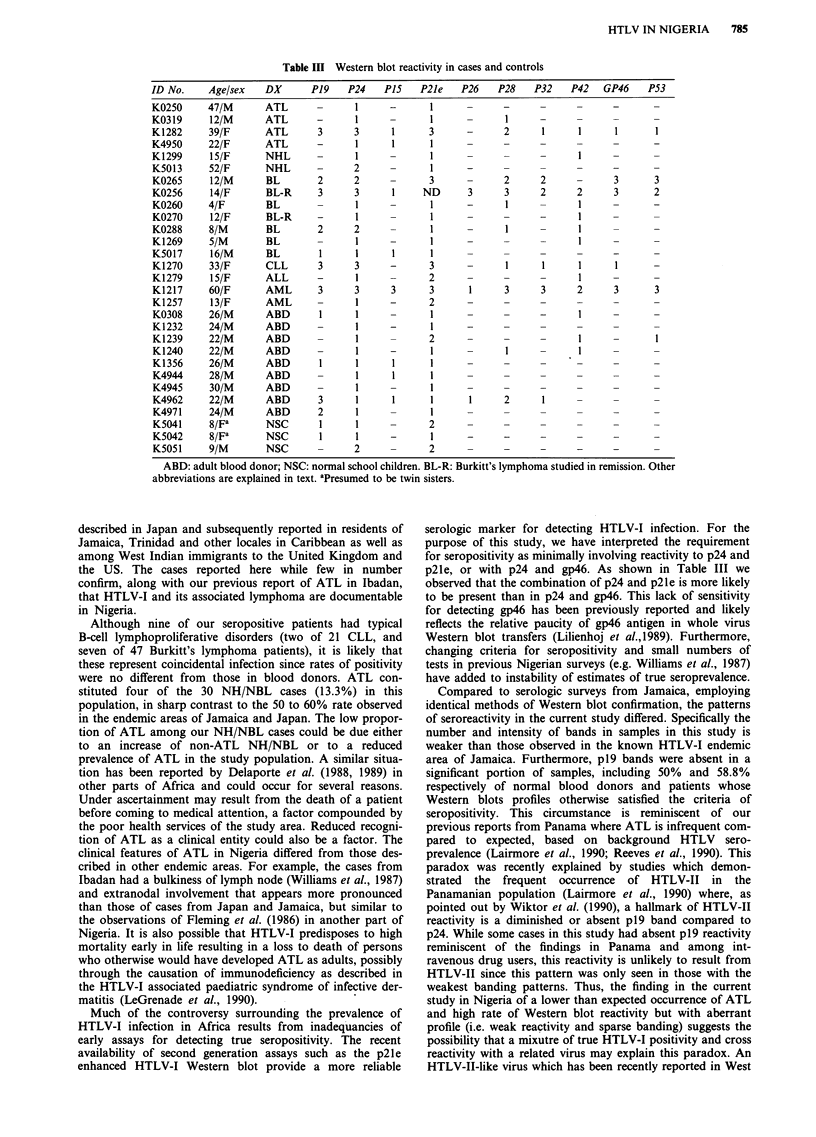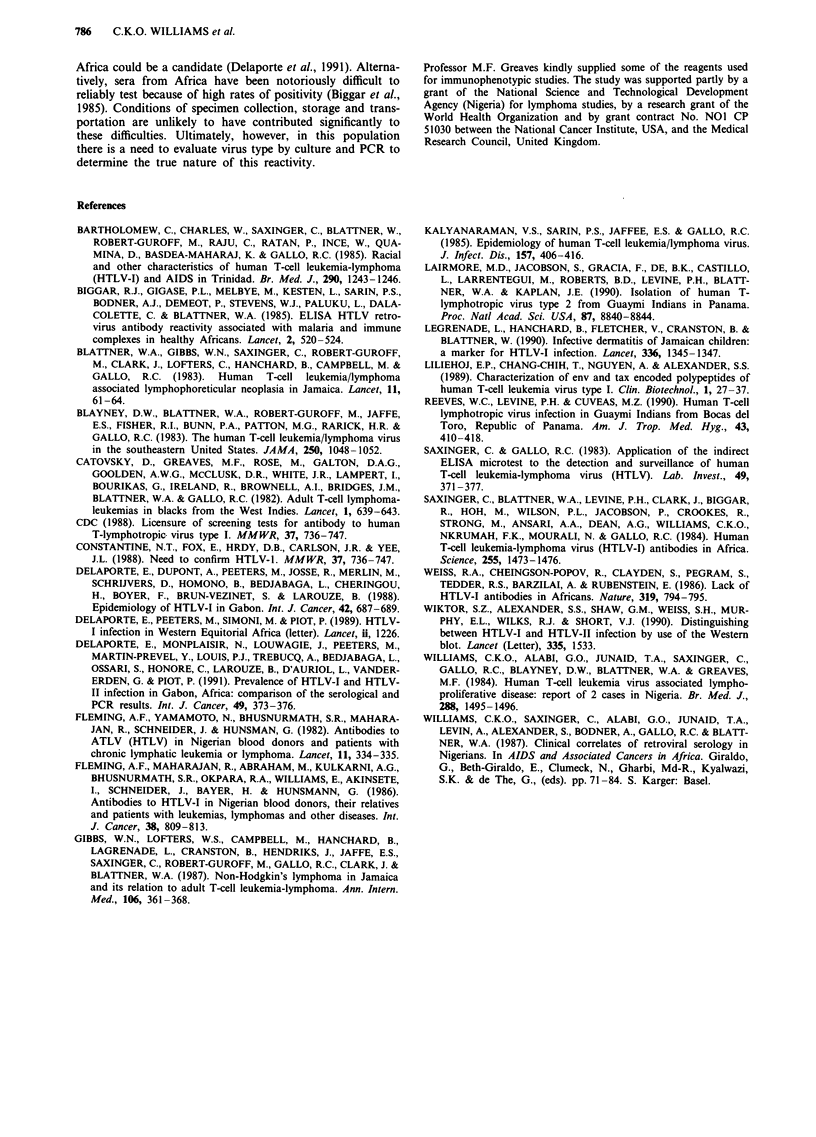# Frequency of adult T-cell leukaemia/lymphoma and HTLV-I in Ibadan, Nigeria.

**DOI:** 10.1038/bjc.1993.142

**Published:** 1993-04

**Authors:** C. K. Williams, S. S. Alexander, A. Bodner, A. Levine, C. Saxinger, R. C. Gallo, W. A. Blattner

**Affiliations:** Department of Haematology, College of Medicine University of Ibadan, Nigeria.

## Abstract

Sera from a small sample of adult blood donors, healthy school children and patients with lymphoma, leukaemia, non-haematologic cancer, congenital and inflammatory disorders from Ibadan, Nigeria were screened for HTLV-I antibody by an enzyme-linked immunoabsorbent assay and confirmed by investigational Western blot. Seventy-nine of 236 positively screened samples could not be tested for confirmation. Seropositive reactivity was observed in nine of 123 blood donors, and 3 of 46 healthy school children but banding patterns on Western blot were often sparse. Among non-Burkitt's non Hodgkin's lymphoma patients six of 30 were HTLV-I positive including four of four with clinical features of adult T-cell leukaemia (ATL). Other clinical conditions had a frequency of positivity indistinguishable from healthy donors. Western blot patterns ranged from strong with multiple bands, which were uncommon, to those with only p24 and p21 envelope positive which were frequent. Given the relative paucity of clinical ATL and the unusual Western blot patterns the true rate of HTLV-I infection may be lower than estimated. It is possible that a cross-reactive HTLV-I-like virus accounts for this pattern.


Br. J. Cancer (1993), 67, 783–786 © Macmillan Press Ltd., 1993

### Frequency of adult T-cell leukaemia/lymphoma and HTLV-I in Ibadan, Nigeria

C.K.O. Williams,[1] S.S. Alexander,[4] A. Bodner,[4] A. Levine,[2] C. Saxinger,[3] R.C. Gallo,[3] & W.A. Blattner[2]

[1]*Department of Haematology, College of Medicine University of Ibadan, Nigeria;* [2]*Viral Epidemiology Section National Cancer Institute, Executive Plaza North, Room 434, 6130 Executive Blvd., Rockville, Maryland 20892;* [3]*Laboratory of Tumor Cell Biology, National Cancer Institute, Building 37, 9000 Rockville Pike, Bethesda, Maryland 20892;* [4]*Biotech Research Laboratories, 1600 East Guide Drive, Rockville, Maryland 20850, USA.*

**Summary** Sera from a small sample of adult blood donors, healthy school children and patients with lymphoma, leukaemia, non-haematologic cancer, congenital and inflammatory disorders from Ibadan, Nigeria were screened for HTLV-I antibody by an enzyme-linked immunoabsorbent assay and confirmed by investigational Western blot. Seventy-nine of 236 positively screened samples could not be tested for confirmation. Seropositive reactivity was observed in nine of 123 blood donors, and 3 of 46 healthy school children but banding patterns on Western blot were often sparse. Among non-Burkitt's non Hodgkin's lymphoma patients six of 30 were HTLV-I positive including four of four with clinical features of adult T-cell leukaemia (ATL). Other clinical conditions had a frequency of positivity indistinguishable from healthy donors. Western blot patterns ranged from strong with multiple bands, which were uncommon, to those with only p24 and p21 envelope positive which were frequent. Given the relative paucity of clinical ATL and the unusual Western blot patterns the true rate of HTLV-I infection may be lower than estimated. It is possible that a cross-reactive HTLV-I-like virus accounts for this pattern.

Human T-cell lymphotropic virus type I (HTLV-I) is endemic in various parts of the world, including the southern coastal areas of Japan, the West Indies and, to a lesser degree, the southeastern region of the United States among people of African descent (Kalyanaraman *et al.*, 1985; Blayney *et al.*, 1983; Catovsky *et al.*, 1982). In Jamaica, an HTLV-I endemic area, between 50% and 70% of all non-Hodgkin's lymphoma cases are HTLV-I seropositive (Blattner *et al.*, 1983; Gibbs *et al.*, 1987). In Trinidad/Tobago, HTLV-I infection is restricted largely to persons of African ancestry despite the fact that the population is equally divided between persons of Asian and African origin supporting the concept that the virus is endemic in Africa (Bartholomew *et al.*, 1985). This hypothesis was supported by our previous case report of a Nigerian with classical adult T-cell leukaemia/lymphoma (Williams *et al.*, 1984) and recent population surveys of HTLV-I in various locales of Africa (Fleming *et al.*, 1982; Saxinger *et al.*, 1984; Fleming *et al.*, 1986; Williams *et al.*, 1987). While the validity of the early HTLV-I serology in Africa has been questioned (Weiss *et al.*, 1986) especially because of the remarkable discrepancy between the enzyme-linked screening and confirmatory assays when compared with the more sensitive Western immunoblot assay (Constantine *et al.*, 1988; CDC, MMWR, 1988), recent advances in technology have significantly improved the accuracy of HTLV diagnosis.

The current study evaluated HTLV in healthy subjects and patients with various lymphoreticular and haematologic disorders as well as other non-haematological neoplasms from Ibadan, Nigeria. Other patients studied included patients with chronic non-neoplastic disorders, including infections, autoimmune and heredity disorders requiring multiple transfusions.

### Methods

All patients attending the hematooncology service at the University College Hospital, Ibadan over a 15 month period were recruited. Eligible disease included non-Hodgkin's, Hodgkin's, and Burkitt lymphoma (BL), acute and chronic lymphocytic leukaemia as well as various hematologic disorders including disorders requiring poly transfusion. One hundred and twenty-three adult blood donors were recruited from the hospital blood bank. Blood was also obtained from 46 Elementary School children resident in a socio-economically deprived area of the city. Aliquots of 1–2 ml of sera from the blood samples were placed in polystyrene shipment tubes and stored for 1–6 months at $-20°C$ prior to shipment in dry ice to the Laboratory of Tumor Cell Biology of the National Cancer Institute, Bethesda, MD, where they were screened for HTLV-I antibodies by a whole virus enzyme-linked immunoassay (ELISA) (Saxinger & Gallo, 1983). Confirmatory Western blot testing was performed using an investigational Western blot assay incorporating the recombinant transmembrane gene product p21e into standard whole virus (Biotech, Inc) (Liliehoj *et al.*, 1989). The seropositivity rates were computed as the product of the probabilities of positive outcome of the two tests. The 95% confidence intervals were computed from these characteristics using a standard statistical methodology.

Several patterns of Western blot results were identified: a multi-band pattern including strong reactivity with the gag antigen p24 and either with the external envelope gp46 and/or a recombinant p21e transmembrane portion of the envelope; an oligo banding pattern, usually p24 and gp21e; an 'indeterminate pattern' where single or multiple bands including both gag and envelope reactivity; and a negative pattern where all bands were absent. Positives included all samples with at least a gag and envelope reactivity. Indeterminants were classified as negative. These criteria of definition of reactivity pattern are similar to those suggested recently (CDC, MMWR, 1988) for confirming blood bank screen positive samples.

In selected cases mononuclear cells recovered from blood, ascitic fluid or teased form tissue biopsy samples were phenotyped with a panel of monoclonal antibodies, including OKT-11A (anti-E-rosette receptor), WT-1 (anti-T), OKT-3, OKT-4, and OKT-8 using indirect immunofluorescence. Adult T-cell leukaemia/lymphoma (ATL) was diagnosed in this study primarily on clinical grounds depending on the presence of, at least, any two of the following characteristic clinical features: hypercalcaemia occurring at any time in the course of the disease, cutaneous involvement, osteolytic

Correspondence: C.K.O. Williams, Allan Blair Memorial Clinic, Saskatchewan Cancer Foundation, 4101 Dewdney Avenue, Regina, Saskatchewan, S4T 7T1 Canada.
Received 30 August 1992; and in revised form 10 November 1992.

lesion and leukaemic peripheral and marrow blood picture. Demonstration of mature and/or helper T-cell phenotype was considered as additional supportive evidence. Similar criteria were applied in the characterisation of ATL in Jamaica (Gibbs *et al.*, 1987).

### Results

In Table I are summarised the results of the serosurvey of various disease states, and the healthy school children and normal donors. There were a total of 30 paitents with non-Hodgkin's, non-Burkitt's lymphoma (NH/NBL) of whom six were confirmed HTLV-I seropositive. Of these cases four had features of ATL and all four were HTLV-I positive. With the exception of one case with borderline hypercalcaemia, none of the remaining 26 cases had features of ATL. Thus, 100% of ATL cases in this survey were HTLV-I seropositive. Otherwise, seropositivity rates among patients with non-ATL NH/NBL, BL, Hodgkin's disease, variants of leukaemia and other haemopoietic malignancies, non-haemopoietic malignancies and chronic non-neoplastic disorders did not differ from those among healthy blood donors (mean age: 23.9 years) or school children (mean age: 8.9 years).

Among ATL cases atypical large mononuclear cells were observed in the bone marrow but not peripheral blood smears of cases K0250, K0319, and K4950 (Table II). Histologic features were those of aggressive lymphoma with 'bizzare' (K0319), plasmacytoid (K0250), centroblastic (K4950) or 'high grade' (K1282) cell types.

In Table II are summarised the clinical features of the four ATL cases and two positive NB/NBL. There were two male and two female ATL cases, the youngest being 12 years old and the oldest 47. One of four had skin involvement, three of three bone marrow involvement and three of four with lytic bone lesions. Two of three tested had hypercalcaemia and two of two tested had T-cell phenotype. Two of three had bulky extranodal involvement. The clinical course of disease was rapid with death usually occurring within weeks of admission. The two positive NH/NBL had not been adequately investigated and did not manifest enough clinical features for the establishment of a diagnosis of ATL. The cases of Burkitt's lymphoma presented with typical clinical and laboratory features of the disease including median age of 5.5 years, small non-cleaved pyroninophilic lymphoid cells (7/7), jaw tumours (5/7) and mesenteric abdominal masses (4/7). The seven seropositive cases consisted of five of 40 (12.5%) consecutive previously untreated BL patients, and two of seven (28.6%) patients studied in remission (BL-R). Summarised in Table III are the Western blot patterns for the cases and normal controls. Four of five positives were females and they represent the only cases where both p21e (transmembrane envelope) and gp46 external envelope reactivity was present. In general the remaining cases had weaker reactivity with an oligo banding pattern present.

### Discussion

The case series reported here confirms previous reports of a strong correlation of HTLV-I to non-Hodgkin's lymphoma patients with features of adult T-cell leukaemia as originally

**Table I** Results of HTLV-I testing in normal donor and patients with neoplastic and non-neoplastic diseases at the University College Hospital, Ibadan, Nigeria

| | No. with ELISA OD ratio < cutoff | No. with ELISA OD[b]ratio > cutoff ± WB confirmed: NEG | IND | POS | ND | Total no. studied | % Sero-positivity[a] | 95% Confidence interval % |
|---|---|---|---|---|---|---|---|---|
| *Lymphoma* | | | | | | | | |
| ATL | 0 | 0 | 0 | 4 | 0 | 4 | 100 | – |
| Non-ATL NH/NBL | 6 | 9 | 2 | 2 | 7 | 26 | 11.5 | 0–42.2 |
| BL | 25 | 8 | 6 | 7 | 1 | 47 | 15.4 | 0–35.4 |
| HD | 7 | 3 | 3 | 0 | 0 | 13 | 0 | – |
| *Cancers* | | | | | | | | |
| Acute leukaemia | 12 | 7 | 0 | 3 | 4 | 26 | 15.9 | 0–48.7 |
| CLL | 13 | 4 | 1 | 2 | 1 | 21 | 10.9 | 0–39.6 |
| CML | 3 | 4 | 1 | 0 | 1 | 9 | 0 | – |
| Others | 11 | 6 | 3 | 2[c] | 6 | 28 | 11.3 | 0–41.1 |
| Non-neoplastic chronic diseases | 11 | 11 | 1 | 3 | 7 | 33 | 13.4 | 0–50.9 |
| *Normal subjects* | | | | | | | | |
| Blood donors | 42 | 29 | 16 | 9 | 27 | 123 | 11.0 | 0–24.8 |
| School children | 14 | 3 | 1 | 3 | 25 | 46 | 21.2 | 0–57.9 |
| Total no. studied | 144 | 84 | 34 | 39 | 79 | 380 | 14.3 | 6.6–24.4 |

NEG: negative. IND: indeterminate. POS: positive. ND: not done. WB: Western blot. [a]See Methods for description of derivation. [b]Optical density. [c]1/3 cases of hepatoma and 1/1 case of Wilm's tumour.

**Table II** Clinical and laboratory features of patients diagnosed clinically to have ATL or who have HTLV-I seropositive malignant lymphoma (excluding Burkitt's lymphoma)

| ID No. | Age/sex | Leukaemia | Skin | BM | Bone | Other site | Serum Ca++ | T-cell phenotype | Diagnosis | HTLV-I WB |
|---|---|---|---|---|---|---|---|---|---|---|
| K0250 | 47/M | No | No | Yes | Jaw | Mouth Floor | 10.0[a] | ND | ATL | POS |
| K0319 | 12/M | No | No | Yes | ND | Liver | 12.8[a] | CD4+ | ATL | POS |
| K1282 | 39/F | No | Yes | ND | Skull | No | ND | ER+; CD2+ | ATL | POS |
| K4950 | 22/F | No | No | Yes | Ilium | ND | 9.3 | ND | ATL | POS |
| K1299 | 15/F | No | No | Yes | ND | ND | 9.8[a] | ND | NHL | POS |
| K5013 | 52/F | No | No | ND | No | No | ND | ND | NHL | POS |

WB: Western blot. ND: not done or not examined. BM: bone marrow. ER: sheep red blood cell receptor. [a]Elevated serum calcium level (in mg $dl^{-1}$).

**Table III** Western blot reactivity in cases and controls

| *ID No.* | *Age/sex* | *DX* | *P19* | *P24* | *P15* | *P21e* | *P26* | *P28* | *P32* | *P42* | *GP46* | *P53* |
|---|---|---|---|---|---|---|---|---|---|---|---|---|
| K0250 | 47/M | ATL | – | 1 | – | 1 | – | – | – | – | – | – |
| K0319 | 12/M | ATL | – | 1 | – | 1 | – | 1 | – | – | – | – |
| K1282 | 39/F | ATL | 3 | 3 | 1 | 3 | – | 2 | 1 | 1 | 1 | 1 |
| K4950 | 22/F | ATL | – | 1 | 1 | 1 | – | – | – | – | – | – |
| K1299 | 15/F | NHL | – | 1 | – | 1 | – | – | – | 1 | – | – |
| K5013 | 52/F | NHL | – | 2 | – | 1 | – | – | – | – | – | – |
| K0265 | 12/M | BL | 2 | 2 | – | 3 | – | 2 | 2 | – | 3 | 3 |
| K0256 | 14/F | BL-R | 3 | 3 | 1 | ND | 3 | 3 | 2 | 2 | 3 | 2 |
| K0260 | 4/F | BL | – | 1 | – | 1 | – | 1 | – | 1 | – | – |
| K0270 | 12/F | BL-R | – | 1 | – | 1 | – | – | – | 1 | – | – |
| K0288 | 8/M | BL | 2 | 2 | – | 1 | – | 1 | – | 1 | – | – |
| K1269 | 5/M | BL | – | 1 | – | 1 | – | – | – | 1 | – | – |
| K5017 | 16/M | BL | 1 | 1 | 1 | 1 | – | – | – | – | – | – |
| K1270 | 33/F | CLL | 3 | 3 | – | 3 | – | 1 | 1 | 1 | 1 | – |
| K1279 | 15/F | ALL | – | 1 | – | 2 | – | – | – | 1 | – | – |
| K1217 | 60/F | AML | 3 | 3 | 3 | 3 | 1 | 3 | 3 | 2 | 3 | 3 |
| K1257 | 13/F | AML | – | 1 | – | 2 | – | – | – | – | – | – |
| K0308 | 26/M | ABD | 1 | 1 | – | 1 | – | – | – | 1 | – | – |
| K1232 | 24/M | ABD | – | 1 | – | 1 | – | – | – | – | – | – |
| K1239 | 22/M | ABD | – | 1 | – | 2 | – | – | – | 1 | – | 1 |
| K1240 | 22/M | ABD | – | 1 | – | 1 | – | 1 | – | 1 | – | – |
| K1356 | 26/M | ABD | 1 | 1 | 1 | 1 | – | – | – | – | – | – |
| K4944 | 28/M | ABD | – | 1 | 1 | 1 | – | – | – | – | – | – |
| K4945 | 30/M | ABD | – | 1 | – | 1 | – | – | – | – | – | – |
| K4962 | 22/M | ABD | 3 | 1 | 1 | 1 | 1 | 2 | 1 | – | – | – |
| K4971 | 24/M | ABD | 2 | 1 | – | 1 | – | – | – | – | – | – |
| K5041 | 8/F[a] | NSC | 1 | 1 | – | 2 | – | – | – | – | – | – |
| K5042 | 8/F[a] | NSC | 1 | 1 | – | 1 | – | – | – | – | – | – |
| K5051 | 9/M | NSC | – | 2 | – | 2 | – | – | – | – | – | – |

ABD: adult blood donor; NSC: normal school children. BL-R: Burkitt's lymphoma studied in remission. Other abbreviations are explained in text. [a]Presumed to be twin sisters.

described in Japan and subsequently reported in residents of Jamaica, Trinidad and other locales in Caribbean as well as among West Indian immigrants to the United Kingdom and the US. The cases reported here while few in number confirm, along with our previous report of ATL in Ibadan, that HTLV-I and its associated lymphoma are documentable in Nigeria.

Although nine of our seropositive patients had typical B-cell lymphoproliferative disorders (two of 21 CLL, and seven of 47 Burkitt's lymphoma patients), it is likely that these represent coincidental infection since rates of positivity were no different from those in blood donors. ATL constituted four of the 30 NH/NBL cases (13.3%) in this population, in sharp contrast to the 50 to 60% rate observed in the endemic areas of Jamaica and Japan. The low proportion of ATL among our NH/NBL cases could be due either to an increase of non-ATL NH/NBL or to a reduced prevalence of ATL in the study population. A similar situation has been reported by Delaporte *et al.* (1988, 1989) in other parts of Africa and could occur for several reasons. Under ascertainment may result from the death of a patient before coming to medical attention, a factor compounded by the poor health services of the study area. Reduced recognition of ATL as a clinical entity could also be a factor. The clinical features of ATL in Nigeria differed from those described in other endemic areas. For example, the cases from Ibadan had a bulkiness of lymph node (Williams *et al.*, 1987) and extranodal involvement that appears more pronounced than those of cases from Japan and Jamaica, but similar to the observations of Fleming *et al.* (1986) in another part of Nigeria. It is also possible that HTLV-I predisposes to high mortality early in life resulting in a loss to death of persons who otherwise would have developed ATL as adults, possibly through the causation of immunodeficiency as described in the HTLV-I associated paediatric syndrome of infective dermatitis (LeGrenade *et al.*, 1990).

Much of the controversy surrounding the prevalence of HTLV-I infection in Africa results from inadequancies of early assays for detecting true seropositivity. The recent availability of second generation assays such as the p21e enhanced HTLV-I Western blot provide a more reliable serologic marker for detecting HTLV-I infection. For the purpose of this study, we have interpreted the requirement for seropositivity as minimally involving reactivity to p24 and p21e, or with p24 and gp46. As shown in Table III we observed that the combination of p24 and p21e is more likely to be present than in p24 and gp46. This lack of sensitivity for detecting gp46 has been previously reported and likely reflects the relative paucity of gp46 antigen in whole virus Western blot transfers (Lilienhoj *et al.*,1989). Furthermore, changing criteria for seropositivity and small numbers of tests in previous Nigerian surveys (e.g. Williams *et al.*, 1987) have added to instability of estimates of true seroprevalence.

Compared to serologic surveys from Jamaica, employing identical methods of Western blot confirmation, the patterns of seroreactivity in the current study differed. Specifically the number and intensity of bands in samples in this study is weaker than those observed in the known HTLV-I endemic area of Jamaica. Furthermore, p19 bands were absent in a significant portion of samples, including 50% and 58.8% respectively of normal blood donors and patients whose Western blots profiles otherwise satisfied the criteria of seropositivity. This circumstance is reminiscent of our previous reports from Panama where ATL is infrequent compared to expected, based on background HTLV seroprevalence (Lairmore *et al.*, 1990; Reeves *et al.*, 1990). This paradox was recently explained by studies which demonstrated the frequent occurrence of HTLV-II in the Panamanian population (Lairmore *et al.*, 1990) where, as pointed out by Wiktor *et al.* (1990), a hallmark of HTLV-II reactivity is a diminished or absent p19 band compared to p24. While some cases in this study had absent p19 reactivity reminiscent of the findings in Panama and among intravenous drug users, this reactivity is unlikely to result from HTLV-II since this pattern was only seen in those with the weakest banding patterns. Thus, the finding in the current study in Nigeria of a lower than expected occurrence of ATL and high rate of Western blot reactivity but with aberrant profile (i.e. weak reactivity and sparse banding) suggests the possibility that a mixutre of true HTLV-I positivity and cross reactivity with a related virus may explain this paradox. An HTLV-II-like virus which has been recently reported in West

Africa could be a candidate (Delaporte *et al.*, 1991). Alternatively, sera from Africa have been notoriously difficult to reliably test because of high rates of positivity (Biggar *et al.*, 1985). Conditions of specimen collection, storage and transportation are unlikely to have contributed significantly to these difficulties. Ultimately, however, in this population there is a need to evaluate virus type by culture and PCR to determine the true nature of this reactivity.

Professor M.F. Greaves kindly supplied some of the reagents used for immunophenotypic studies. The study was supported partly by a grant of the National Science and Technological Development Agency (Nigeria) for lymphoma studies, by a research grant of the World Health Organization and by grant contract No. NO1 CP 51030 between the National Cancer Institute, USA, and the Medical Research Council, United Kingdom.

### References

BARTHOLOMEW, C., CHARLES, W., SAXINGER, C., BLATTNER, W., ROBERT-GUROFF, M., RAJU, C., RATAN, P., INCE, W., QUAMINA, D., BASDEA-MAHARAJ, K. & GALLO, R.C. (1985). Racial and other characteristics of human T-cell leukemia-lymphoma (HTLV-I) and AIDS in Trinidad. *Br. Med. J.*, **290**, 1243–1246.

BIGGAR, R.J., GIGASE, P.L., MELBYE, M., KESTEN, L., SARIN, P.S., BODNER, A.J., DEMEOT, P., STEVENS, W.J., PALUKU, L., DALACOLETTE, C. & BLATTNER, W.A. (1985). ELISA HTLV retrovirus antibody reactivity associated with malaria and immune complexes in healthy Africans. *Lancet*, **2**, 520–524.

BLATTNER, W.A., GIBBS, W.N., SAXINGER, C., ROBERT-GUROFF, M., CLARK, J., LOFTERS, C., HANCHARD, B., CAMPBELL, M. & GALLO, R.C. (1983). Human T-cell leukemia/lymphoma associated lymphophoreticular neoplasia in Jamaica. *Lancet*, **11**, 61–64.

BLAYNEY, D.W., BLATTNER, W.A., ROBERT-GUROFF, M., JAFFE, E.S., FISHER, R.I., BUNN, P.A., PATTON, M.G., RARICK, H.R. & GALLO, R.C. (1983). The human T-cell leukemia/lymphoma virus in the southeastern United States. *JAMA*, **250**, 1048–1052.

CATOVSKY, D., GREAVES, M.F., ROSE, M., GALTON, D.A.G., GOOLDEN, A.W.G., McCLUSK, D.R., WHITE, J.R., LAMPERT, I., BOURIKAS, G., IRELAND, R., BROWNELL, A.I., BRIDGES, J.M., BLATTNER, W.A. & GALLO, R.C. (1982). Adult T-cell lymphoma-leukemias in blacks from the West Indies. *Lancet*, **1**, 639–643.

CDC (1988). Licensure of screening tests for antibody to human T-lymphotropic virus type I. *MMWR*, **37**, 736–747.

CONSTANTINE, N.T., FOX, E., HRDY, D.B., CARLSON, J.R. & YEE, J.L. (1988). Need to confirm HTLV-1. *MMWR*, **37**, 736–747.

DELAPORTE, E., DUPONT, A., PEETERS, M., JOSSE, R., MERLIN, M., SCHRIJVERS, D., HOMONO, B., BEDJABAGA, L., CHERINGOU, H., BOYER, F., BRUN-VEZINET, S. & LAROUZE, B. (1988). Epidemiology of HTLV-I in Gabon. *Int. J. Cancer*, **42**, 687–689.

DELAPORTE, E., PEETERS, M., SIMONI, M. & PIOT, P. (1989). HTLV-I infection in Western Equitorial Africa (letter). *Lancet*, **ii**, 1226.

DELAPORTE, E., MONPLAISIR, N., LOUWAGIE, J., PEETERS, M., MARTIN-PREVEL, Y., LOUIS, P.J., TREBUCQ, A., BEDJABAGA, L., OSSARI, S., HONORE, C., LAROUZE, B., D'AURIOL, L., VANDERERDEN, G. & PIOT, P. (1991). Prevalence of HTLV-I and HTLV-II infection in Gabon, Africa: comparison of the serological and PCR results. *Int. J. Cancer*, **49**, 373–376.

FLEMING, A.F., YAMAMOTO, N., BHUSNURMATH, S.R., MAHARAJAN, R., SCHNEIDER, J. & HUNSMAN, G. (1982). Antibodies to ATLV (HTLV) in Nigerian blood donors and patients with chronic lymphatic leukemia or lymphoma. *Lancet*, **11**, 334–335.

FLEMING, A.F., MAHARAJAN, R., ABRAHAM, M., KULKARNI, A.G., BHUSNURMATH, S.R., OKPARA, R.A., WILLIAMS, E., AKINSETE, I., SCHNEIDER, J., BAYER, H. & HUNSMANN, G. (1986). Antibodies to HTLV-I in Nigerian blood donors, their relatives and patients with leukemias, lymphomas and other diseases. *Int. J. Cancer*, **38**, 809–813.

GIBBS, W.N., LOFTERS, W.S., CAMPBELL, M., HANCHARD, B., LAGRENADE, L., CRANSTON, B., HENDRIKS, J., JAFFE, E.S., SAXINGER, C., ROBERT-GUROFF, M., GALLO, R.C., CLARK, J. & BLATTNER, W.A. (1987). Non-Hodgkin's lymphoma in Jamaica and its relation to adult T-cell leukemia-lymphoma. *Ann. Intern. Med.*, **106**, 361–368.

KALYANARAMAN, V.S., SARIN, P.S., JAFFEE, E.S. & GALLO, R.C. (1985). Epidemiology of human T-cell leukemia/lymphoma virus. *J. Infect. Dis.*, **157**, 406–416.

LAIRMORE, M.D., JACOBSON, S., GRACIA, F., DE, B.K., CASTILLO, L., LARRENTEGUI, M., ROBERTS, B.D., LEVINE, P.H., BLATTNER, W.A. & KAPLAN, J.E. (1990). Isolation of human T-lymphotropic virus type 2 from Guaymi Indians in Panama. *Proc. Natl Acad. Sci. USA*, **87**, 8840–8844.

LEGRENADE, L., HANCHARD, B., FLETCHER, V., CRANSTON, B. & BLATTNER, W. (1990). Infective dermatitis of Jamaican children: a marker for HTLV-I infection. *Lancet*, **336**, 1345–1347.

LILIEHOJ, E.P., CHANG-CHIH, T., NGUYEN, A. & ALEXANDER, S.S. (1989). Characterization of env and tax encoded polypeptides of human T-cell leukemia virus type I. *Clin. Biotechnol.*, **1**, 27–37.

REEVES, W.C., LEVINE, P.H. & CUVEAS, M.Z. (1990). Human T-cell lymphotropic virus infection in Guaymi Indians from Bocas del Toro, Republic of Panama. *Am. J. Trop. Med. Hyg.*, **43**, 410–418.

SAXINGER, C. & GALLO, R.C. (1983). Application of the indirect ELISA microtest to the detection and surveillance of human T-cell leukemia-lymphoma virus (HTLV). *Lab. Invest.*, **49**, 371–377.

SAXINGER, C., BLATTNER, W.A., LEVINE, P.H., CLARK, J., BIGGAR, R., HOH, M., WILSON, P.L., JACOBSON, P., CROOKES, R., STRONG, M., ANSARI, A.A., DEAN, A.G., WILLIAMS, C.K.O., NKRUMAH, F.K., MOURALI, N. & GALLO, R.C. (1984). Human T-cell leukemia-lymphoma virus (HTLV-I) antibodies in Africa. *Science*, **255**, 1473–1476.

WEISS, R.A., CHEINGSON-POPOV, R., CLAYDEN, S., PEGRAM, S., TEDDER, R.S., BARZILAI, A. & RUBENSTEIN, E. (1986). Lack of HTLV-I antibodies in Africans. *Nature*, **319**, 794–795.

WIKTOR, S.Z., ALEXANDER, S.S., SHAW, G.M., WEISS, S.H., MURPHY, E.L., WILKS, R.J. & SHORT, V.J. (1990). Distinguishing between HTLV-I and HTLV-II infection by use of the Western blot. *Lancet* (Letter), **335**, 1533.

WILLIAMS, C.K.O., ALABI, G.O., JUNAID, T.A., SAXINGER, C., GALLO, R.C., BLAYNEY, D.W., BLATTNER, W.A. & GREAVES, M.F. (1984). Human T-cell leukemia virus associated lymphoproliferative disease: report of 2 cases in Nigeria. *Br. Med. J.*, **288**, 1495–1496.

WILLIAMS, C.K.O., SAXINGER, C., ALABI, G.O., JUNAID, T.A., LEVIN, A., ALEXANDER, S., BODNER, A., GALLO, R.C. & BLATTNER, W.A. (1987). Clinical correlates of retroviral serology in Nigerians. In *AIDS and Associated Cancers in Africa*. Giraldo, G., Beth-Giraldo, E., Clumeck, N., Gharbi, Md-R., Kyalwazi, S.K. & de The, G., (eds). pp. 71–84. S. Karger: Basel.